# Functional Analysis of A Novel Splicing Mutation in The Mutase Gene of Two Unrelated Pedigrees

**DOI:** 10.22074/cellj.2016.4568

**Published:** 2016-08-24

**Authors:** Somayeh Ahmadloo, Saeed Talebi, Mohammad Miryounesi, Parvin Pasalar, Mohammad Keramatipour

**Affiliations:** 1 Department of Medical Genetics, School of Medicine, Tehran University of Medical Sciences, Tehran, Iran; 2 Genomic Research Center, Shahid Beheshti University of Medical Sciences, Tehran, Iran; 3 Department of Biochemistry, School of Medicine, Tehran University of Medical Sciences, Tehran, Iran; 4 Endocrinology and Metabolism Research Center, Endocrinology and Metabolism Clinical Sciences Institute, Tehran University of Medical Sciences, Tehran, Iran

**Keywords:** Amino Acid Metabolism, Inborn Errors, Methylmalonyl-CoA Mutase, 3´ Splice Acceptor Site

## Abstract

**Objective:**

Methylmalonic acidura (MMA) is a rare autosomal recessive inborn error of metabolism. In this study we present a novel nucleotide change in the mutase (MUT) gene of two unrelated Iranian pedigrees and introduce the methods used for its functional analysis.

**Materials and Methods:**

Two probands with definite diagnosis of MMA and a common novel variant in the *MUT* were included in a descriptive study. Bioinformatic prediction of the splicing variant was done with different prediction servers. Reverse transcriptionpolymerase chain reaction (RT-PCR) was done for splicing analysis and the products were analyzed by sequencing.

**Results:**

The included index patients showed elevated levels of propionylcarnitine (C3). Urine organic acid analysis confirmed the diagnosis of MMA, and screening for mutations in the *MUT* revealed a novel C to G variation at the 3´ splice acceptor site in intron 12. In silico analysis suggested the change as a mutation in a conserved sequence. The splicing analysis showed that the C to G nucleotide change at position -3 in the acceptor splice site can lead to retention of the intron 12 sequence.

**Conclusion:**

This is the first report of a mutation at the position -3 in the *MUT* intron 12 (c.2125-3C>G). The results suggest that the identified variation can be associated with the typical clinical manifestations of MMA.

## Introduction

Methylmalonic acidura (MMA, MIM# 251000) is one of the inborn errors of metabolism. This rare organic acid metabolism disorder is inherited in an autosomal recessive manner and occurs with the estimated prevalence rates ranging between 1:48,000 (1) and 1:100,000 (2). MMA is often caused by either deficiency of the enzyme methylmalonylCoA mutase (MCM) or a defect in the biosynthesis of its cofactor, adenosyl-cobalamin (AdoCbl). It is important to note that the presence of cofactor AdoCbl causes catalyzing the isomerization of methylmalonyl-CoA into succinyl-CoA by the mitochondrial enzyme methylmalonyl-CoA mutase. Therefore, defects either in the apoenzyme or in biosynthesis of its cofactor cause abnormal accumulation of methylmalonate in the body fluids (3). Affected individuals are typically present in the newborn period with ketoacidosis, vomiting, and failure to thrive (4). These patients often suffer from significant anorexia, which may require periods of prolonged nasogastric feeding, and are below the mean for height (5). 

The human MCM enzyme is encoded by the mutase gene (*MUT*), mapped to chromosome 6p12.3, that is composed of 13 exons. The *MUT* open reading frame consists of 2.7 kb, which encods 750 amino acids. The leader sequence, which consists of the first 32 residues, directs the precursor proapoenzyme into the mitochondria. Following the entree into the mitochondria and removal of the leader sequence, the remaining protein sequence consists of two distinct functional domains: a substrate-binding site (residues 88-422), and a C-terminal vitamin B12-binding domain (residues 578-750). The C-terminal domain contains the enzyme active site (3,6,8). 

Molecular analysis of the genes involved in inherited metabolic diseases have characterized the mutational spectrum, identifying splicing defects as the second most frequent type of mutation, after the missense type (9). Point mutations in the intronic regions near the splice junctions can affect mRNA splicing, altering the resultant RNA sequence, which could have a profound impact on protein expression (10). It is important to note that at least 200 mutations in *MUT* have been identified (11). In addition, a number of ethnic-specific mutations have been identified in Caucasian and Asian patients (12). 

In this study we introduce a novel single nucleotide variation (SNV) at position -3 of the *MUT* acceptor splice site in intron 12 in two Iranian MMA patients from independent families. This is the first report demonstrating that this particular nucleotide change in the conserved region of the *MUT* can be associated with typical clinical manifestations of MMA. 

## Materials and Methods

### Subjects and clinical assessment

The research was a descriptive study and approved by a duly constituted Ethics Committee of the Tehran University of Medical Sciences (Tehran, Iran). The parents provided informed consent. 

In this study two index patients from unrelated families with definite diagnosis of MMA which had a common novel variant in the 3´ splice acceptor site of *MUT* 12^th^ intron were included. Clinical evaluations included standard history, physical examination and metabolic profiling of acylcarnitine, amino-acids and organic acids. Screening for *MUT* variants were done in a diagnostic laboratory (Centogene, Iran). 

### Bioinformatic predictions

Frequency of the variant was looked over different population databases including dbSNP, HGMD, the 1 k human genome, the ESP6500 and Exome Variant Server. 

The effect of nucleotide change on the splicing at the acceptor site, was evaluated by NetGen2 software from CBS prediction services and Human Splice Finder (HSF) system (13,15). The scaled Combined Annotation Dependent Depletion (CADD) score (scaled C-sore) (16) and MutationTaster (17) were used to evaluate the disease causing effects of the variation. 

PhastCons and PhyloP values presented in the MutationTaster were used for conservation analysis. In addition, sequences flanking the acceptor site of *MUT* intron 12 in 32 different species were checked and aligned with the proposed human sequence in the Ensembl Genome Browser. 

### DNA and RNA extractions and cDNA synthesis

After obtaining an informed consent and receiving the peripheral blood samples from both parents (Family #1), genomic DNA was isolated using standard phenol/chlorophorm extraction and ethanol precipitation method (18). Total RNA was also extracted from a normal control and one parent using whole blood samples and Hybrid-RTM Blood RNA isolation kit (GeneAll Biotechnology Co., Ltd, South Korea) following manufacturer’s instructions. Synthesis of cDNA was performed with 500 ng of total RNA (Solis BioDyne Co., Tartu, Estonia). Gene specific reverse primer and random hexamer were used for priming. 

### Polymerase chain reaction and reverse transcription-polymerase chain reaction

In order to confirm the carrier pattern in parents, specific primers (MCM-EX13 primer pair) were used (4) for amplification of exon 13 and its flanking sequences (Table 1). The amplification conditions for the genomic DNA of parents in order to check their heterozygosity consisted of 40 cycles in which denaturation occurred at 95˚C for 30 seconds, annealing at 60˚C for 30 seconds, and extension at 72˚C for 30 seconds. 

For RNA splicing analysis a specific forward primer was designed in the junctions of exons 10 and 11 (MUT-EX10-11 primer), in order to not amplify DNA sequences (Table 1). In addition, two specific reverse primers were designed: one in intron 12 (MUT-I12 primer) and one in exon 13 (MUT-EX13 primer). The specificity of primers was analyzed with the cDNA from HSFPI Ш cell line that expresses *MUT*. RT-PCR was done with primers designed for normal variant and the quality of cDNA was checked with β-actin primers in Table 1 (19). For amplification of the normal variant, the annealing temperature was at 55˚C for 30 seconds and extension at 72˚C for 1 minute. For the aberrant variant, the annealing and extension temperatures were set at 50˚C for 30 seconds and at 72˚C for 1 minute, respectively. All products were run on 1% agarose gels, followed by dideoxy sequencing by capillary electrophoresis (Pishgam. Biotech Company, Iran). 

## Results

### Proband in family #1 

**Fig.1:**
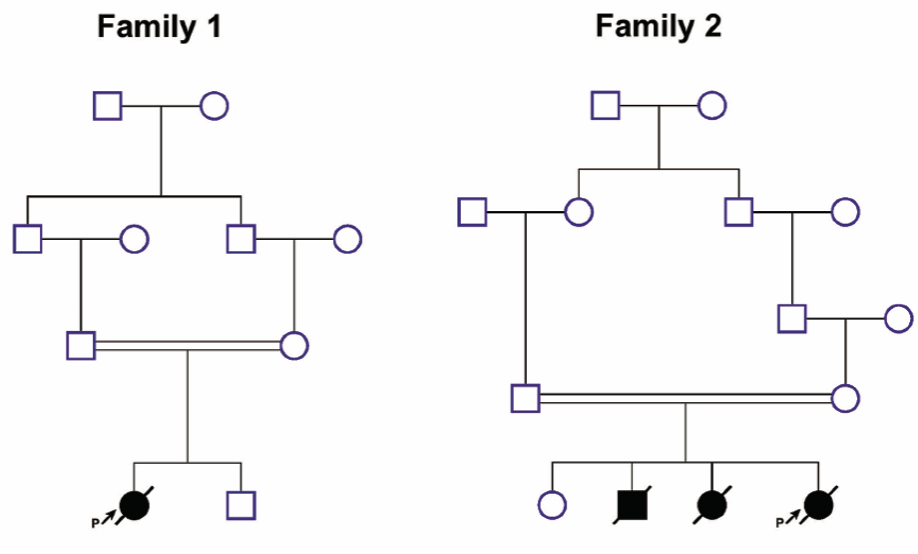
Pedigrees of the two Iranian families with MMA. As the figure shows, in family no.1, the proband had a healthy sibling. By contrast, in the second family, the proband had two affected and one healthy siblings. MMA; Methylmalonic aciduria and P; Proband.

The proband girl was born in the 37^th^ week of gestation from a 27-years-old mother. The parents were first degree cousins (Fig.1, left pedigree). At birth, she was admitted into NICU because of asphyxia and respiratory distress due to meconium aspiration. She also experienced seizure on day 4 of life. Her developmental milestones were within the acceptable range until the age of 7 months, when she experienced a metabolic attack with acidosis and swallowing problems with reflux and recurrent vomiting. Afterwards, her developmental milestones regressed. Neurological examination showed a mild to moderate central hypotonia with peripheral hypertonia and moderate global developmental delay. The brain magnetic resonance imaging (MRI) in the 7^th^ month of age showed mild atrophy with abnormal signals in basal ganglia and substantia nigra. The death occurred at the age of 3 years old following an episode of metabolic acidosis.

Evaluation of acylcarnitine profile on dry blood spot showed elevated C3 level (C3=9.8 µmol/l, the reference level for the respective age is <5 µmol/l). The organic acid test results confirmed diagnosis of MMA according to the pronounced increase in methylmalonate excretion with increased methylcitrate excretion in urine. Screening for mutations in the *MUT* gene revealed a novel homozygous C to G SNV at position -3 of the acceptor splice site in intron 12 (NG_007100.1: g.36281C>G; NG_007100.1 (MUT_v001): c.2125- 3C>G; chr6:49399572G>C). 

**Table 1:** List of DNA primers used


Primer names	Oligonucleotide sequence (5´-3´)	Primer length (mer)	Annealing temperature (˚C)

MCM-EX13	F: ATTTCCGGTAAAATGGAAAATAGTGGC	27	55
	R: CCAAACACTTCTCAATATCATCAAGCA	27	55
MUT-EX10-11	F: TCTGCTATCAAGAGGGTTCA	20	50
MUT-I12	R: CTATCATTACTCAAGATTCCCA	22	55
MUT-EX13	R: CTCAATATCATCAAGCACCTG	21	50
Beta actin	F: AGCCTCGCCTTTGCCGA	17	62
	R: CTGGTGCCTGGGGCG	15	60


### Proband in family #2

The proband girl was born in the 38th week of gestation from a 31-years-old mother. The parents were first cousins once removed (Fig.1, right pedigree). This couple had two children who died with the same disease as the proband. There was no history of either neurologic disease (including intellectual disabilities), abortion, dead infant or child, or any other specific disorder in family. The first episode of disease occurred 3 days after birth with severe vomiting, hypotonia, and metabolic acidosis. In the first year of life, there were 4 episodes of metabolic acidosis. There was no history of seizure reported in this patient.

The patient had a mild developmental delay. In the 18th month of life, brain MRI showed a normal appearance. The death occurred at the age of 3.5 years old following an episode of metabolic acidosis. Evaluation of acylcarnitine profile on dry blood spot in this patient also showed a significant increased C3 level (C3=46.22 µmol/l, the reference level for age is <2.5 µmol/l). Screening for mutations in the *MUT* demonstrated a homozygous C to G nucleotide variation at the acceptor splice site in intron 12 (c.2125-3C>G).

In the Table 2, the clinical and laboratory findings in probands are compared to the common findings in patients with methylmalonic aciduria (20).

**Table 2:** Clinical and laboratory findings in patients with methylmalonic aciduria compared to the probands MMA (Infancy)


	MMA (Infancy)	Proband in family 2	Proband in family 1

Failure to thrive	+	+	NA
Vomiting	+	+	+
Anorexia	+	+	+
Hypotonicity	+	+	+
Acidosis	+	+	+
Glucose (B)	Low to NL	NL	NA
Ammonia (B)	NL to High	NL	NA
Organic acids (U): Methylmalonate, methylcitrate	High	High	High
Lactate	NL to High	High	NA


NL; Normal, NA; Not available, and MMA; Methylmalonic acidura.

### In silico analysis (first step)

This variant was not observed and has not been reported in the 1 k human genome, HGMD, the ESP6500, dbSNP and Exome Variant Server databases. 

**Fig.2:**
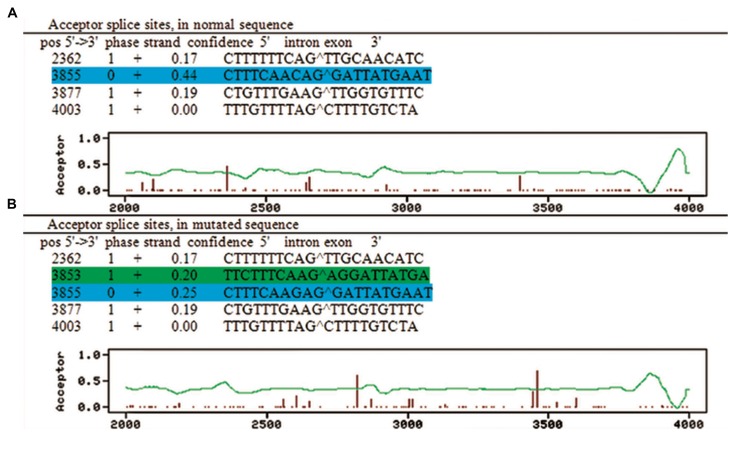
In silico acceptor splice site prediction in the normal and mutated sequences. A. The level of confidence for the the normal acceptor splice site in the normal sequence (relative to the cutoff used to find nearly all true sites) is 0.44 (highlighted in blue) and B. The level of confidence for the the normal acceptor splice site in the mutated sequence decreased to 0.25 (highlighted in blue) and a new splice site with the confidence level of 0.20 appears in the 3' side of the mutated nucleotide (highlighted in green).

The in silico analysis by NetGen2 showed that after C nucleotide substitution by G at -3 position, while the probability of main splice site decreases, the probability of an alternative splice site increases (Fig.2). Position weight (HSF) matrices predicted that the substitution of C to G-allele resulted in loss of 3´ splice donor site with decreasing the consensus value from 93.38 to 83.08. Likewise, the maximum entropy algorithm predicted that the score was significantly reduced from 9.23 to -3.2 for motif sequence with mutant G-allele. 

The scaled CADD score (scaled C-sore) was more than 15, therefore the variation was also classified as a deleterious variation (Phred-scaled Cscore v1.3 = 15.51). The MutationTaster also classified the nucleotide change as a disease causing variation and also predicted the increased probability for new splicing sites. 

**Fig.3:**
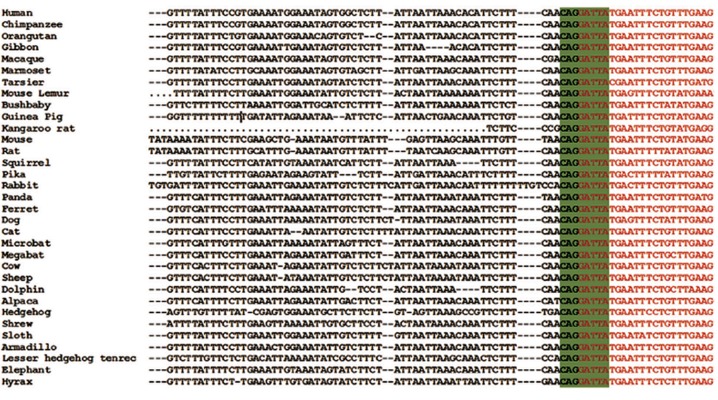
Alignment of the conserved region within *MUT* intron 12 in different species. The exon 13 sequence is presented with italic and underlined fromatting. The highly conserved octa-nucleotide sequence (CAGGATTA) is highlighted. The first nucleotide (C) in this conserved region is the locus of mutation.

The orthologous sequences flanking the acceptor site of *MUT* intron 12 in 32 different species allowed us to verify and ensure that the highlighted sequences are conserved (Fig.3). In addition, the conservation analysis by PhastCons and PhyloP methods indicated a highly conserved nucleotide region (PhyloP value=3.412, PhastCons value=1). 

### Carrier testing of parents (second step)

**Fig.4:**
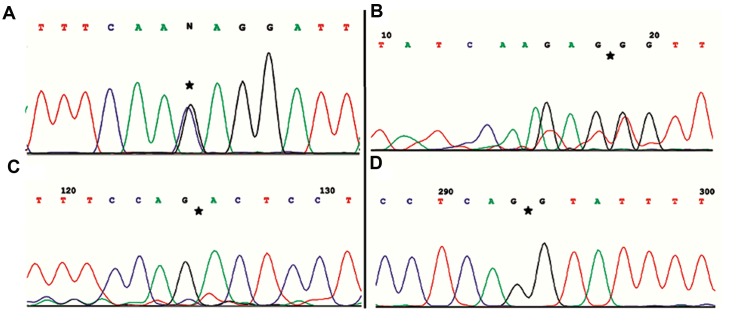
Sequencing chromatograms of the proband’s father in the first family. A. Heterozygote variant (star) in DNA sequence using MCMEX13 reverse primer, B. Exons 10-11 junction sequence (star) using MUT-I12 primer, C. Exons 11-12 junction sequence (star) using MUT-EX10-11 primer, and D. The junctional sequence of exon 12 and intron 12 (star) using MUT-EX10-11 primer.

In the first step for the functional analysis, DNA analysis for both parents confirmed a heterozygote substitution at position c.2125-3 C>G (with respect to the coding sequence; NM_000255.3), which is near the splice acceptor site in exon 13 (Fig.4A). 

### RNA splicing analysis (third step)

**Fig.5:**
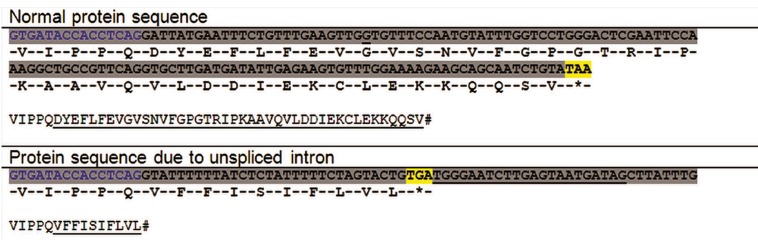
Normal and deduced aberrant proteins. The normal mRNA sequence related to the distal part of exon 12 (blue nucleotides) and the proximal part of exon 13 (other nucleotides) is presented in the upper part. The protein sequence related to the exon 13 is underlined. The aberrant mRNA sequence related to the distal part of exon 12 and proximal part of intron 12 is presented in the lower part. The deduced protein sequence related to the intron 12 is underlined. The underlined nucleotides show the MUT-I12-R primer position which is after the predicted premature stop codon.

The normal variant (using primer pair of C and E) was expressed from normal controls and heterozygote parents but the aberrant variant (using primer pair of C and D) was expressed only in the heterozygote parents. The sequencing of the products amplified with both primer pairs confirmed that the nucleotide change interferes with the splicing of the intron 12 (Fig.4B-D) and that the new splice site predicted by in silico analysis is not functional. The aberrant variant forms a truncated protein, which compromises 718 amino acids and has an abnormal C-terminal. Interestingly, intron 12 retention causes addition of 10 amino acids to the protein and causes a premature stop codon leading to deletion of exon 13. As a result, the aberrant variant interrupts the vitamin B12 binding site. The predicted premature stop codon is located within the amplified sequence by primers A and D (Fig.5). 

## Discussion

In the present study, we have shown that a novel point mutation at position -3 in the *MUT* acceptor splice site conserved sequence can result in the inclusion of the complete upstream intron. This appears to be a rare event. The absence of variation in population databases and consistency with co-segregation within the pedigrees suggests a pathogenic role for this nucleotide substitution. In addition the mRNA splicing analysis successfully ruled out the predicted new splicing sites. 

The invariant 5´-GT and 3´-AG are not the sole determinants of accurate splicing, as the extensive consensus sequence spanning 5´ and 3´ splice sites is also a determining factor (21). A comprehensive survey, scanning 400 vertebrates, has determined the " ^-4^N ^-3^C_78_^-2^A_100_^-1^G_100_/ ^+1^G_55_" consensus sequence in the 3´ splice sites (22). It is important to note that the most frequent nucleotide at position of -3 of the acceptor splice site is C. The second prioritized base is either A or T, and the least frequent is G (23). 

The Novel mutation reported here leads to retention of the intron 12, which adds 10 amino acids to the encoding protein after exon 12 but also causes a premature stop codon leading to the complete deletion of the following exon 13. This mutation interrupts the vitamin B12 binding site. Thus, it renders the truncated protein incapable of performing its biological function. 

It should be noted that mutations in the splice sites of introns can have numerous consequences, which cannot be predicted by the primary sequence alone. Other less well-defined factors such as secondary pre-mRNA structure or mRNA-protein interactions may also play a role. It is well-established that the splicing reaction does not proceed sequentially along the precursor RNA and rather it follows a preferred pathway. Mutations in the splice sites can disturb this order (24). 

According to the joint consensus recommendation of the American College of Medical Genetics and Genomics and the Association for Molecular Pathology for the interpretation of sequence variants, the strategies used for analysis of the novel variant in this study had effectively demonstrated its pathogenicity (25): i. The demonstrated deleterious effect due to retention of the intron 12 in the well-established functional study provided an strong evidence for pathogenicity (PS3), ii. The absence of the variant in population databases made a moderate evidence of pathogenicity (PM2), iii. Protein length changing based on computational and predictive data also provided a moderate evidence for pathogenicity (PM4), iv. Multiple lines of computational evidence supported a deleterious effect on the gene product (PP3), v. Cosegregation with disease in two unrelated family made a supporting evidence for pathogenicity (PP1), and vi. The clinical and paraclinical findings in proband patients which were highly specific for MMA due to the *MUT* defect also provided a supporting evidence for pathogenicity (PP4). 

Finally due to one strong, two moderate and three supporting evidences for the pathogenicity this variant was classified as a pathogenic variant. 

## Conclusion

This study presented a mutation at the position -3 in the *MUT* intron 12 (c.2125-3C>G) for the first time. The strategies introduced for the functional analysis in this study suggested that the identified variation can be associated with the typical clinical manifestations of MMA. However the exact functional consequences of this particular mutation requires further studies. 
